# Combination therapy of itraconazole and an acylhydrazone derivative (D13) for the treatment of sporotrichosis in cats

**DOI:** 10.1128/spectrum.03967-23

**Published:** 2024-04-22

**Authors:** Isabella Dib Ferreira Gremião, Gabriela Reis Pereira-Oliveira, Sandro Antonio Pereira, Maria Lopes Corrêa, Luana Pereira Borba-Santos, Alessandra Lifsitch Viçosa, Ashna Garg, Krupanandan Haranahalli, Deveney Dasilva, Nivea Pereira de Sa, Gabriel S. Matos, Vanessa Silva, Cristina Lazzarini, Caroline Mota Fernandes, Kildare Miranda, Jhon Jhamilton Artunduaga Bonilla, Anna Letícia Nunes, Leonardo Nimrichter, Iwao Ojima, John Mallamo, John B. McCarthy, Maurizio Del Poeta

**Affiliations:** 1 Laboratory of Clinical Research on Dermatozoonoses in Domestic Animals, Evandro Chagas National Institute of Infectious Diseases, Rio de Janeiro, Brazil; 2 Laboratory of Fungal Cell Biology, Carlos Chagas Filho Institute of Biophysics, Rio de Janeiro, Brazil; 3 Laboratory of Experimental Pharmacotechnics, Institute of Drug Technology – Farmanguinhos, Oswaldo Cruz Foundation (Fiocruz), Rio de Janeiro, Brazil; 4 Institute of Chemical Biology and Drug Discovery, Stony Brook University, Stony Brook, New York, USA; 5 Department of Chemistry, Stony Brook University, Stony Brook, New York, USA; 6 Department of Microbiology and Immunology, Stony Brook University, Stony Brook, New York, USA; 7 Laboratory of Cellular Ultrastructure Hertha Meyer, Carlos Chagas Filho Institute of Biophysics and National Center for Structural Biology and Bioimaging, Federal University of Rio de Janeiro, Rio de Janeiro, Brazil; 8 Laboratory of Eukaryotic Glycobiology (LaGE), Instituto de Microbiologia Paulo de Góes, Universidade Federal do Rio de Janeiro, Rio de Janeiro, Brazil; 9 MicroRid Technologies Inc., Dix Hills, New York, USA; 10 Division of Infectious Diseases, School of Medicine, Stony Brook University, Stony Brook, New York, USA; 11 Veterans Administration Medical Center, Northport, New York, USA; Universidade de Sao Paulo, Sao Paulo, Brazil

**Keywords:** cats, *Sporothrix*, sporotrichosis, itraconazole, terbinafine, acylhydrazone, anti-fungal therapy, fungal infection

## Abstract

Acylhydrazone (AH) derivatives represent a novel category of anti-fungal medications that exhibit potent activity against *Sporothrix* sp., both *in vitro* and in a murine model of sporotrichosis. In this study, we demonstrated the anti-fungal efficacy of the AH derivative D13 [4-bromo-*N*′-(3,5-dibromo-2-hydroxybenzylidene)-benzohydrazide] against both planktonic cells and biofilms formed by *Sporothrix brasiliensis*. In a clinical study, the effect of D13 was then tested in combination with itraconazole (ITC), with or without potassium iodide, in 10 cats with sporotrichosis refractory to the treatment of standard of care with ITC. Improvement or total clinical cure was achieved in five cases after 12 weeks of treatment. Minimal abnormal laboratory findings, e.g., elevation of alanine aminotransferase, were observed in four cats during the combination treatment and returned to normal level within a week after the treatment was ended. Although highly encouraging, a larger and randomized controlled study is required to evaluate the effectiveness and the safety of this new and exciting drug combination using ITC and D13 for the treatment of feline sporotrichosis.

**IMPORTANCE:**

This paper reports the first veterinary clinical study of an acylhydrazone anti-fungal (D13) combined with itraconazole against a dimorphic fungal infection, sporotrichosis, which is highly endemic in South America in animals and humans. Overall, the results show that the combination treatment was efficacious in ~50% of the infected animals. In addition, D13 was well tolerated during the course of the study. Thus, these results warrant the continuation of the research and development of this new class of anti-fungals.

## INTRODUCTION

Sporotrichosis is a mycotic disease that affects humans and animals and is caused by pathogenic species of the genus *Sporothrix*, such as *Sporothrix schenckii* and *Sporothrix brasiliensis*. It is the most common subcutaneous mycosis (1). *S. brasiliensis* and *S. schenckii* have been described as the main worldwide causal agents of mycosis in cats (2). However, in South America, and particularly in Brazil, *S. brasiliensis* is the most prevalent species (3, 4), and in certain Asian countries, *S. schenckii* is the most common species (5).

The cat is considered the most susceptible animal to *Sporothrix* infection, presenting various clinical forms, such as single skin lesion, multiple skin lesions, ulcers, or/and disseminated systemic infection. These clinical manifestations are often not responsive to treatment (6–9). The treatment of sporotrichosis in cats is an important measure of disease control not only to reduce the animal burden but also to diminish or/and prevent transmission from cats to humans (10). However, treatment is often protracted for a long time (weeks or months), and because of this long treatment, cat owner compliance is low. In addition, the fungus can also become refractory to the treatment. As a result, cats do not respond well to the standard of care, resulting in therapeutic failure (6, 11–14).

Few effective anti-fungal agents are available for treating feline sporotrichosis, and most of them are often used as monotherapy regimens (15). The standard of care (SOC) includes itraconazole (ITC) alone or is associated with potassium iodide (KI), and this regime is the most common therapeutic regimen in Brazil (16, 17). Ketoconazole, sodium iodide, fluconazole, amphotericin B (AMB), terbinafine, posaconazole, local heat therapy, cryosurgery, and surgical removal of the lesions have also been described for treating sporotrichosis in cats, but their efficacy varies greatly (16). A recent study shows for the first time that nikkomycin Z could be an option for the treatment of sporotrichosis caused by *S. brasiliensis* (18), although this treatment regime will be quite expensive.

ITC is a triazole with a broad spectrum of anti-fungal activity that blocks the synthesis of ergosterol (major sterol component of fungal plasma membranes) through the inhibition of the fungal cytochrome P450-dependent enzyme lanosterol 14-a-demethylase (19). However, the decrease of ergosterol synthesis leads to a fungal growth arrest and not to cell death. As such, ITC is fungistatic, and therapeutic failure with ITC is common (13). Thus, the testing of new and fungicidal anti-fungal agents (alone or in combination with ITC) is warranted (20).

The family of acylhydrazones (AHs) is a new class of anti-fungal agent molecules which target the vesicular transport and cell cycle progression of fungi and indirectly impact glucosylceramide synthesis (21). As a result, toxic sphingolipids accumulate, such as sphingosine, leading to mitochondria and vesicular damage (21). Thus, acylhydrazone compounds are highly fungicidal and able to kill fungi within a few hours (22). An acylhydrazone known as D13 [4-bromo-*N*′-(3,5-dibromo-2-hydroxybenzylidene)-benzohydrazide] (ChemBridge ID# 5475098) showed strong anti-fungal activity with very low toxicity in mammalian cells (22). Using a checkerboard assay, studies have shown that D13 improved the efficacy of commercially available drugs against various fungi. Against *S. brasiliensis*, D13 promoted yeast disruption and ultrastructural changes (23). Pharmacokinetic studies of D13 were performed in a murine model and were very promising with high oral availability (22). Importantly, D13 exhibited strong activity against different strains of *Sporothrix in vitro* when used alone or in combination with ITC (23). The D13 anti-fungal effectiveness was further displayed in a murine model of sporotrichosis (23).

Thus, we performed a preliminary clinical study using D13 in cats affected by sporotrichosis. The study was approved only as a “compassionate drug use,” meaning D13 could only be administered after the SOC failed. As such, it was not possible to have a control group in which D13 was not administered. Out of 10 cats that were enrolled, 4 cats were clinically cured and 1 significantly improved. Thus, our results are promising and encouraging the design of a larger clinical study using D13 as a primary therapy with the inclusion of a control group.

## RESULTS

### Enrollment

This study was a pilot clinical study. The authors did not inflict sporotrichosis to cats. Cats affected with sporotrichosis were taken by their owner to the Laboratory of Clinical Research on Dermatozoonoses in Domestic Animals at Oswaldo Cruz Foundation (Fiocruz), in Rio de Janeiro, Brazil, to seek diagnosis and treatment. Upon confirming the diagnosis of sporotrichosis, cats were treated using the SOC. They mainly received ITC alone or in combination with KI (Table 1). One cat (case 2) also received terbinafine (TRB). If the cat did not respond to the SOC (see below), a possibility to add D13 was proposed to the owner of the cat as compassionate drug use. If the owner agreed, D13 was added to the therapeutic protocol.

**TABLE 1:** Clinical information and summary table of the 10 cases of sporotrichosis treated with the experimental drug acylhydrazone D13 in various combinations with the standard of care^*b*^

Case	Sex	Age(mo)	Wt (kg)	Con	Lesions	Resp signs	Prior treatment	New ther prot	Treat time (wk)	LAB	Out
1	M	24	2.9	S	Cut and nasal muc	Dys	ITC (12 wk) ITC + KI (12 wk)	ITC + KI + D13	32	↑ALT	Cure
2	M	24	4.1	G	Cut and nasal muc	Dys	ITC + KI (4 wk) ITC + TRB (8 wk)	ITC + KI + TRB + D13	24	↑ALT	Cure
3	M	12	4.3	G	Cut and nasal muc	Sneezing, nasal dis	ITC (12 wk)	ITC + D13	12	↑ALT	Cure
4^*a*^	M	24	4.9	G	Cut	None	ITC (4 wk)	ITC + D13	12	None	Cure
5	M	41	4.2	G	Nasal muc	Dys, nasal dis	ITC (4 wk)	ITC + D13	4	↑ALT	Imp/failure
6	M	48	4.5	G	Cut and nasal muc	Dys, nasal dis	ITC (8 wk)	ITC + D13	12	None	Aband
7	M	24	3.8	G	Cut and nasal muc	Nasal dis	ITC + KI (2 wk)	ITC + D13	8	None	Failure
8	M	24	4.5	S	Cut	None	ITC (16 wk) ITC + KI (2 wk)	ITC + KI + D13	8	None	Aband
9	F	24	3.5	S	Cut and nasal muc	Dys, sneezing	ITC + KI (8 wk)	ITC + KI + D13	72	None	Death
10^*a*^	F	24	3.1	S	Cut and nasal muc	Dys, sneezing, nasal dis	ITC + KI (24 wk)	ITC + KI + D13	<1	None	Aband

^*a*^ The clinical isolates from these two cats were identified as *Sporothrix brasiliensis,* but after the identification, these clinical samples were lost and no longer available for minimum inhibitory concentration and synergistic testing. All other clinical isolates were available (see Tables 1 and 2).

^*b*^ Aband, abandonment; ALT, alanine aminotransferase; con, health condition at the initiation of the standard of care; cut, cutaneous; dis, discharge; dys, dyspnea; F, female; G, good; imp, improved; ITC, itraconazole; KI, potassium iodine; LAB; laboratory testing (blood work); M, male; muc, mucosal; out, outcome; resp, prot, protocol; respiratory; S, serious; ther, therapeutic; TRB, terbinafine; treat, treatment.

**TABLE 2:** Anti-*Sporothrix* activity of D13 and ITC against clinical isolates recovered from eight cats^*a*^^,*b*^

Clinical isolates	D13 (µg/mL)		ITC (µg/mL)	
	MIC_50_	MIC_90_	MIC_50_	MIC_90_
Cat 1	0.25	1.0	0.5	1.0
Cat 2	0.12	0.5	0.5	1.0
Cat 3	0.5	8.0	32.0	˃32.0
Cat 5	1.0	4.0	8.0	˃32.0
Cat 6	0.25	2.0	4.0	32.0
Cat 7	2.0	8.0	32.0	˃32.0
Cat 8	1.0	4.0	4.0	32.0
Cat 9	4.0	8.0	8.0	32.0

^*a*^ ITC, itraconazole; MIC_50_ and MIC_90_, minimum inhibitory concentration inhibiting 50% or 90% of growth compared to the growth observed in the absence of drug, respectively.

^*b*^ Tests were performed at least twice.

### *In vitro* anti-fungal activity

Upon clinical suspicion of sporotrichosis, swab samples were collected and placed onto Sabouraud-dextrose agar. Plates were incubated for up to 5 days at room temperature (~25C), fungal growth was monitored and fungal identification was performed by standard methods. Fungal isolates were tested for *in vitro* susceptibility against ITC and D13. From the 10 cats, only 8 clinical isolates were obtained. Two isolates (from cases 4 and 10) were highly contamined by bacteria and were lost.

D13 displayed high efficacy in inhibiting the growth of all tested isolates (Table 2), with MIC_50_ ranging from 0.12 to 4.0 μg/mL. As for ITC, only two clinical isolates (cases 1 and 2) showed low MIC_50_ of 0.5 µg/mL. All other isolates had higher MIC_50_ ranging from 4 to 32 μg/mL (Table 2), suggesting that these isolates were, in general, more resistant to ITC.

### Synergistic studies

Because D13 was intended to be administered solely as a combination therapy, we assessed the synergist effect of D13 when combined with ITC. Synergistic activity was examined using the checkerboard method and the Bliss method. Using the checkerboard assay, the combination of D13 and ITC resulted in synergistic activity or in no interaction (Table 3). Using the Bliss method, we found that the combination of D13 and ITC resulted in having additive activity in all tested clinical isolates (Table 3).

**TABLE 3:** Synergistic activity illustrated as FICi of itraconazole combined with D13 against *sporothrix* clinical isolated from cats^*a*^^,*b*^

Clinical isolates	Checkerboard method		Bliss method	
	Ʃ FICi	Activity	Score	Activity
Cat 1	0.65 ± 0.12	Indifferent	1.72	Additive
Cat 2	0.52 ± 0.01	Indifferent	−7.98	Additive
Cat 3	0.98 ± 0.04	Indifferent	−7.43	Additive
Cat 5	0.66 ± 0.10	Indifferent	−9.12	Additive
Cat 6	0.68 ± 0.16	Indifferent	−5.98	Additive
Cat 7	0.32 ± 0.07	Synergistic	−8.56	Additive
Cat 8	1.01 ± 0.02	Indifferent	−0.38	Additive
Cat 9	0.29 ± 0.03	Synergistic	−8.79	Additive

^*a*^ For the checkerboard method: synergistic drug interactions were identified as synergistic when the FICi was less than 0.5, no interaction or indifferent when the FICi was between 0.5 and 4.0, and antagonistic interactions when the FICi exceeded 4.0. For the Bliss method, scores greater than 10 denote a synergistic interaction; scores ranging from −10 to 10 indicate an additive interaction; and scores below −10 denote an antagonistic interaction. Data represent geometric means ± standard deviations of at least three independent experiments.

^*b*^ FIC, fractional inhibitory concentration index.

### D13 is effective against *S. brasiliensis* biofilm

Recognizing the crucial role of biofilm as an important reservoir of fungal cells, which become more resistant to anti-fungals, we examined the effect of D13 on biofilm formation by *Sporothrix* on a cat’s claw. This is particularly important because this fungus is frequently transmitted from cats to humans by scratches. If *Sporothrix* forms biofilm in the cat’s claw, its potential for transmission is significantly increased.

**Fig 1:**
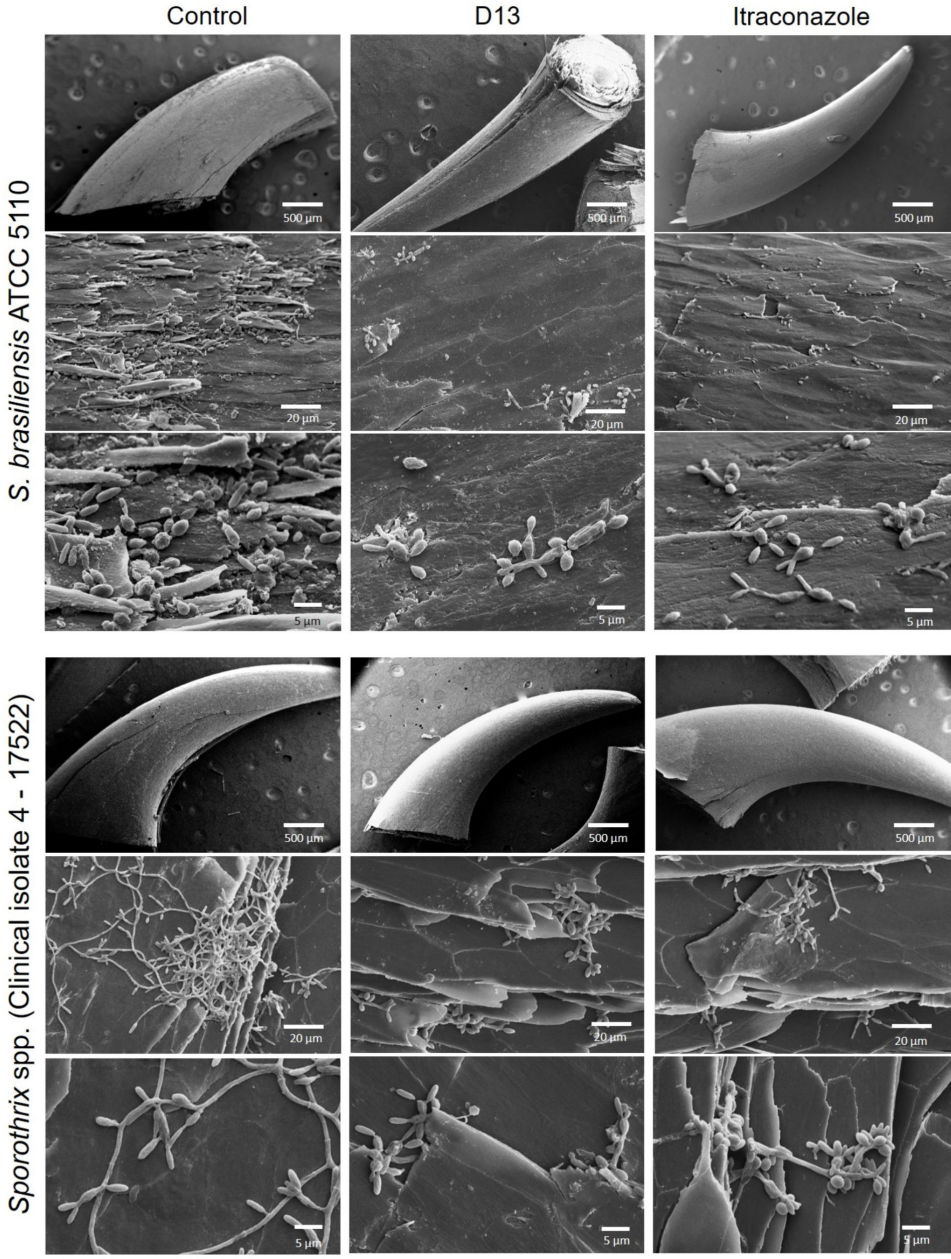
Effect of D13 on *sporothrix* Biofilm. *Sporothrix* yeasts were cultured in RPMI 1640 medium together with cat’s claw fragments and incubated at 37°C for 3 days. Following this, the claws were washed 3 x and subsequently treated with 10 µg/mL of D13 or ITC for another 3 days before being prepared for SEM analysis. In the biofilm control groups, both the ATCC strain of *S. brasiliensis* and the clinically isolated (C.I 17522) strain displayed abundant yeasts and hyphae with a consistently uniform and intact surface. In contrast, cells from both strains treated with either D13 or ITC exhibited slight growth and irregularities on their surfaces.

**Fig 2:**
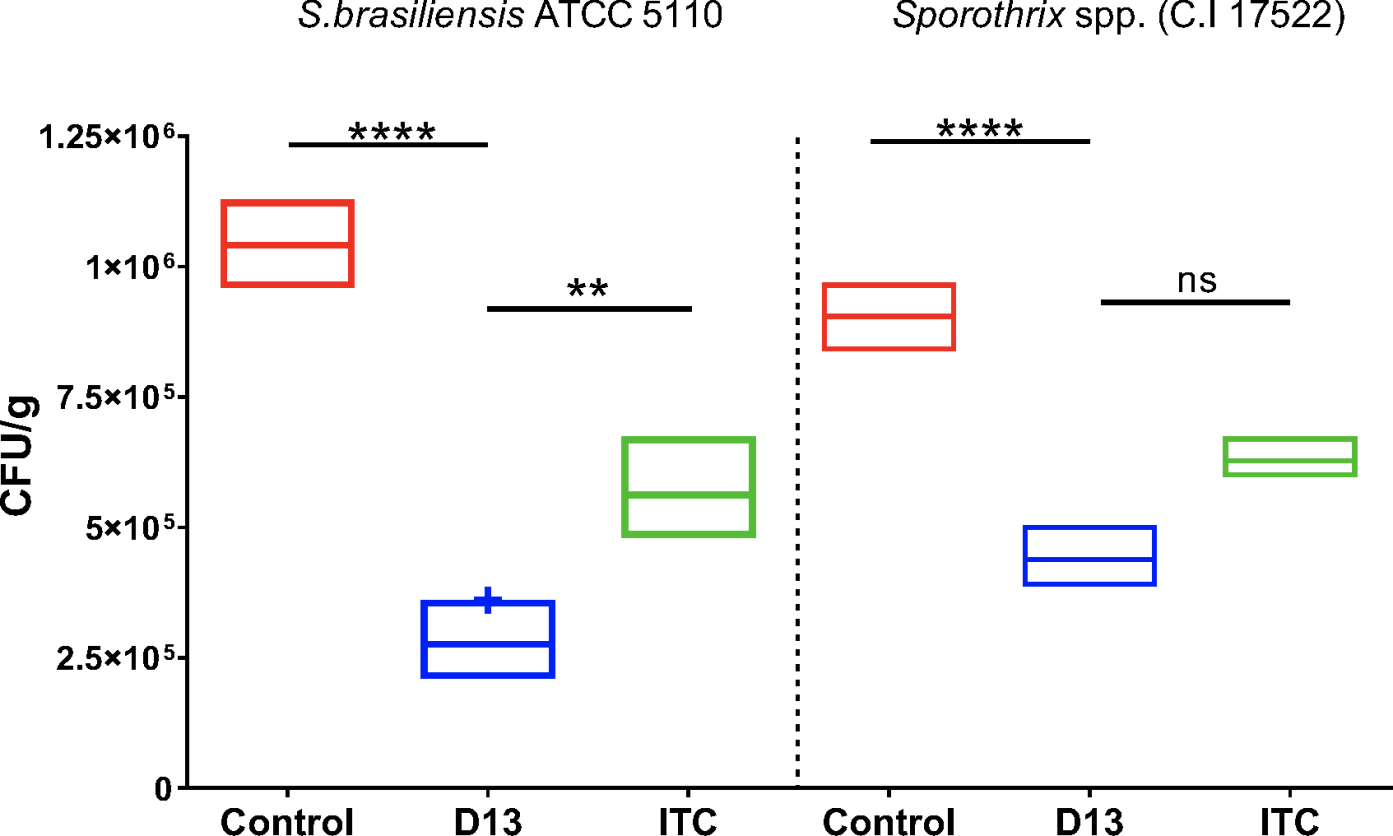
Fungal load of *Sporothrix* biofilm on cat claw fragments. Mature *Sporothrix* biofilms grown for 3 days were treated with D13 or ITC at 10 µg/mL. After 3 days of incubation, the claws were washed and suspended in RMPI/PenStrep 1%. Claws were sonicated and vortexed to drop the cells from the biofilm. Aliquots were placed on brain heart infusion plates and incubated at 37°C for up to 3 days. Floating bars represent the mean minimum to maximum values of three independent experiments. Statistical analysis was performed by one-way analysis of variance and Sidak’s multiple comparison test. **P < 0,0028, ****P < 0,0001. ns, not significant.

For this experiment, we used a reference clinical strain, *Sporothrix brasiliensis* American Type Culture Collection (ATCC) 5110/MYA4823, and a clinical isolate, 4–17522, obtained from our worse case (case 1). The untreated biofilm exhibited a surface densely populated, mostly composed of intact yeasts, as shown in Fig. 1. In contrast, the biofilm treated with D13 showed a substantial reduction in fungal growth, as clearly evidenced by scanning electron microcopy (SEM) images captured at different magnifications (Fig. 1). Analysis of the colony forming unit (CFU) of the tissue confirmed a significant decrease in fungal load in the D13-treated biofilm compared to the control group (*P* < 0.0001) (Fig. 2). The effect of D13 was similar against the referent strain and against the clinical isolate.

Most importantly, treatment with D13 exhibited a pronounced reduction in fungal load when compared to the biofilm treated with ITC (*P* < 0.05) (Fig. 1 and 2), providing further confirmation that D13 appears to be more effective than ITC in inhibiting biofilm formation and growth of *S. brasiliensis*.

### Cases histories

Based on the compelling results obtained in the previous study conducted by our group (21–24), as well as on the promising *in vitro* data observed in this study, we conducted a clinical study using D13 in cats affected with sporotrichosis. The study enrolled a total of 10 cats. All cases included were confirmed as sporotrichosis by isolation of *Sporothrix* spp. in culture from skin ulcers, and S. *brasiliensis* was identified in two cases (cases 1 and 3).

#### Case 1

**Fig 3:**
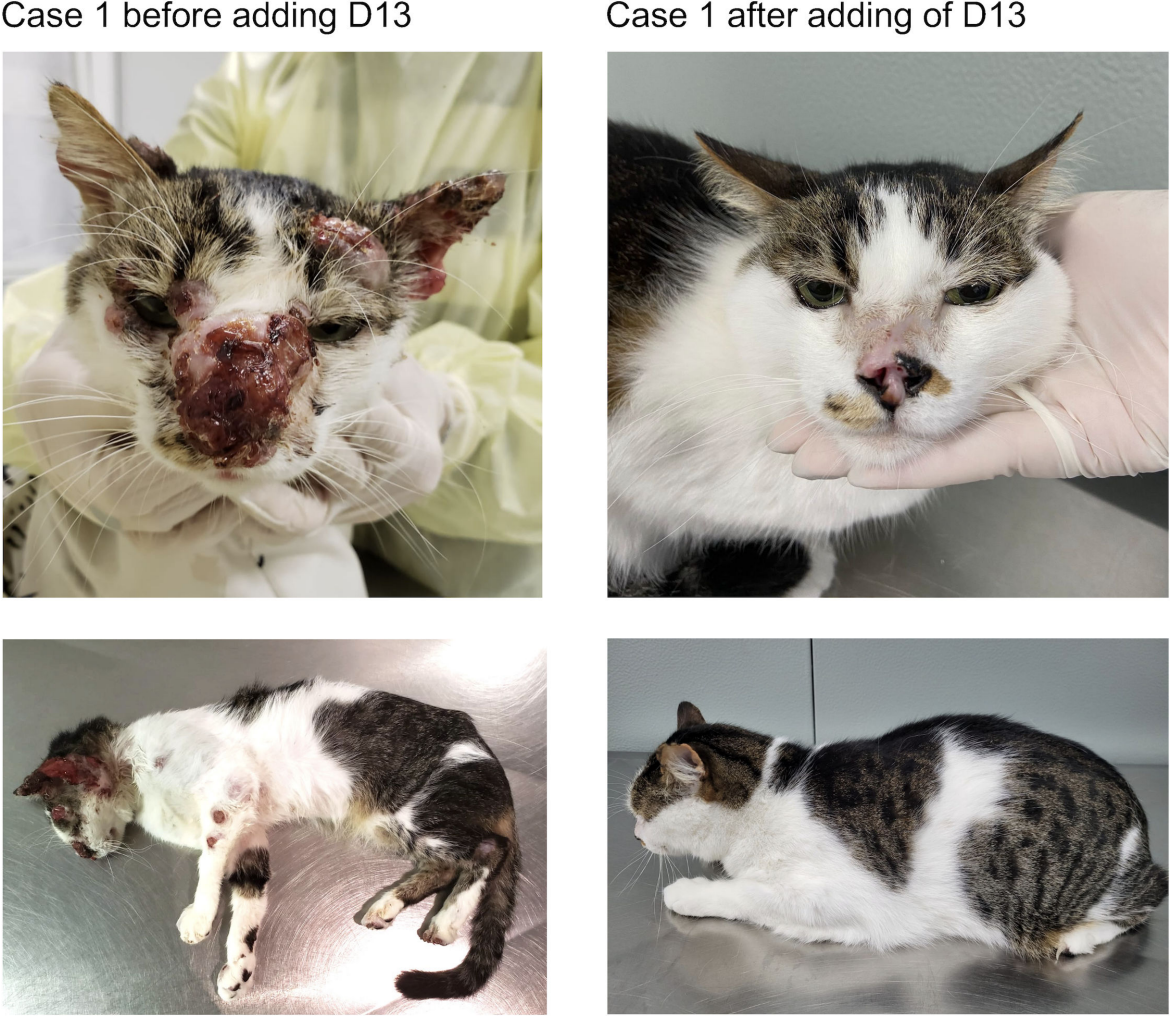
Case 1. The cat presented no improvement of the cutaneous lesions on the head and on the thoracic limbs upon treatment with ITC and KI. After adding D13, the cat significantly improved, and clinical cure was achieved after 8 months of treatment with ITC, KI, and D13 combination

The patient was a 4-year-old neutered male crossbred cat weighing 1.6 kg. The cat was being already treated with ITC (100 mg/cat/day) for 8 months before referral. Clinical examination revealed multiple ulcerated, cutaneous lesions and nodules distributed over the head, nasal region, hind and forelimbs, swelling of both nostrils, dyspnea, and loss of the normal nasal planum architecture. The cat was in serious overall condition and presented hyporexia. Due to poor clinical response, KI (2.5 mg/kg/day) was initiated, in combination with ITC in a lower dose (25 mg/cat/day). Oral sylimarin (30 mg/kg/day) was also prescribed. After 1 month, the dose of ITC was increased up to 50 mg/cat/day as the animal gained weight, while KI and sylimarin were maintained at the same dose. Nevertheless, the cutaneous and nasal mucosal lesions persisted after 3 months of ITC and KI therapy. Thus, D13 (20 mg/kg/day) was added to the ITC (50 mg/cat/day) and KI (2.5 mg/kg/day) therapeutic regimen. Follow-up clinical examinations and analysis of hematological and biochemical parameters, such as urea, creatinine, alanine transaminase (ALT), and aspartate aminotransferase (AST), were performed monthly by the reference center for fungal diseases in animals in Rio de Janeiro. After 3 months of treatment, hyporexia had been noted. After 14 days, mild elevations in ALT (ALT 130.4 U/L, reference interval 6–83 U/L) and AST (AST 93.9 U/L, reference interval 10–80 U/L) were noted. These laboratory abnormalities in ALT and AST levels were transient and returned to the pre-treatment levels when measured at day 28 of treatment. The clinical cure occurred after 8 months of combination therapy (Fig. 3).

#### Case 2

**Fig 4:**
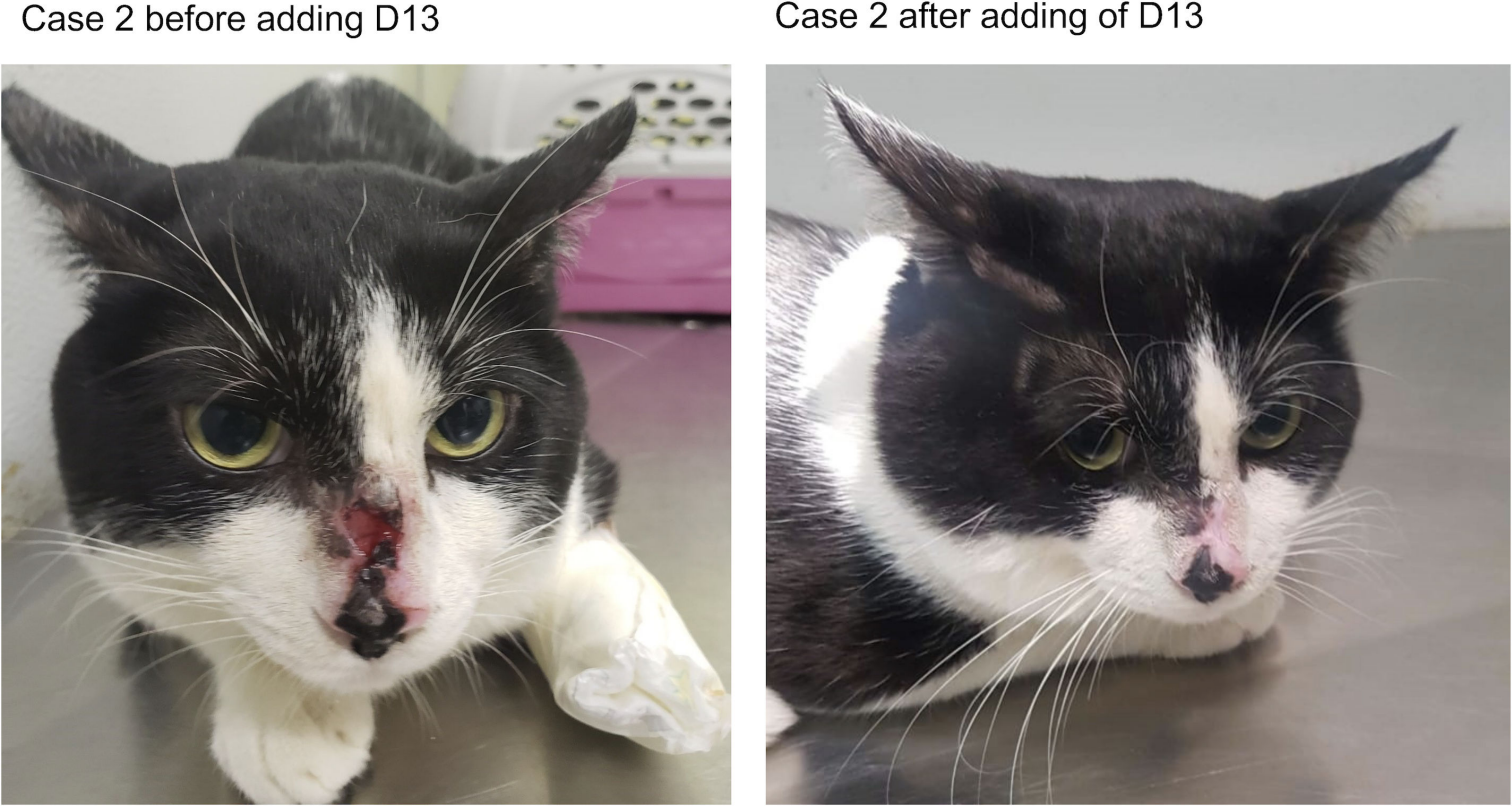
Case 2. The cat presented no improvement of the cutaneous and nasal mucosal lesions upon treatment with ITC, TRB, and KI. After adding D13, the cat significantly improved and clinical cure was achieved after 12 weeks of treatment with ITC, TRB, KI, and D13 combination.

The patient was a 1-year-old non-neutered male crossbred cat weighing 4.1 kg, examined at a reference center for fungal diseases in animals in Rio de Janeiro, Brazil. The cat presented ulcerated, cutaneous lesions on the nasal bridge, lips, left ear, and right forelimb. Swelling of both nostrils, sneezing, nasal discharge, and enlarged mandibular lymph nodes were also observed. Its overall condition was good. Upon identification of *S. brasiliensis*, the cat was treated with ITC (100 mg/cat/day). After 12 weeks of therapy, hyporexia was noted, and no improvement of any cutaneous lesion was observed. In addition, during ITC treatment, three new lesions appeared: one on the nasal bridge near the other lesion and the others two on the right pinna. This indicated a therapeutic failure. Thus, D13 (20 mg/kg/day) was added to ITC (100 mg/cat/day). During the 12 weeks of combination therapy, no clinical adverse reactions were observed. However, mild-to-moderate elevations in ALT between day 30 (ALT 103 U/L, reference interval 10–83 U/L) and day 60 (ALT 266 U/L, reference interval 10–83 U/L) were noted. This abnormality in ALT level was transient and returned to the pre-treatment level when measured at day 90 of treatment (25). The clinical cure (complete healing of the skin lesions) occurred after 6 months of therapy, when the cat was discharged (Fig. 4).

#### Case 3

**Fig 5:**
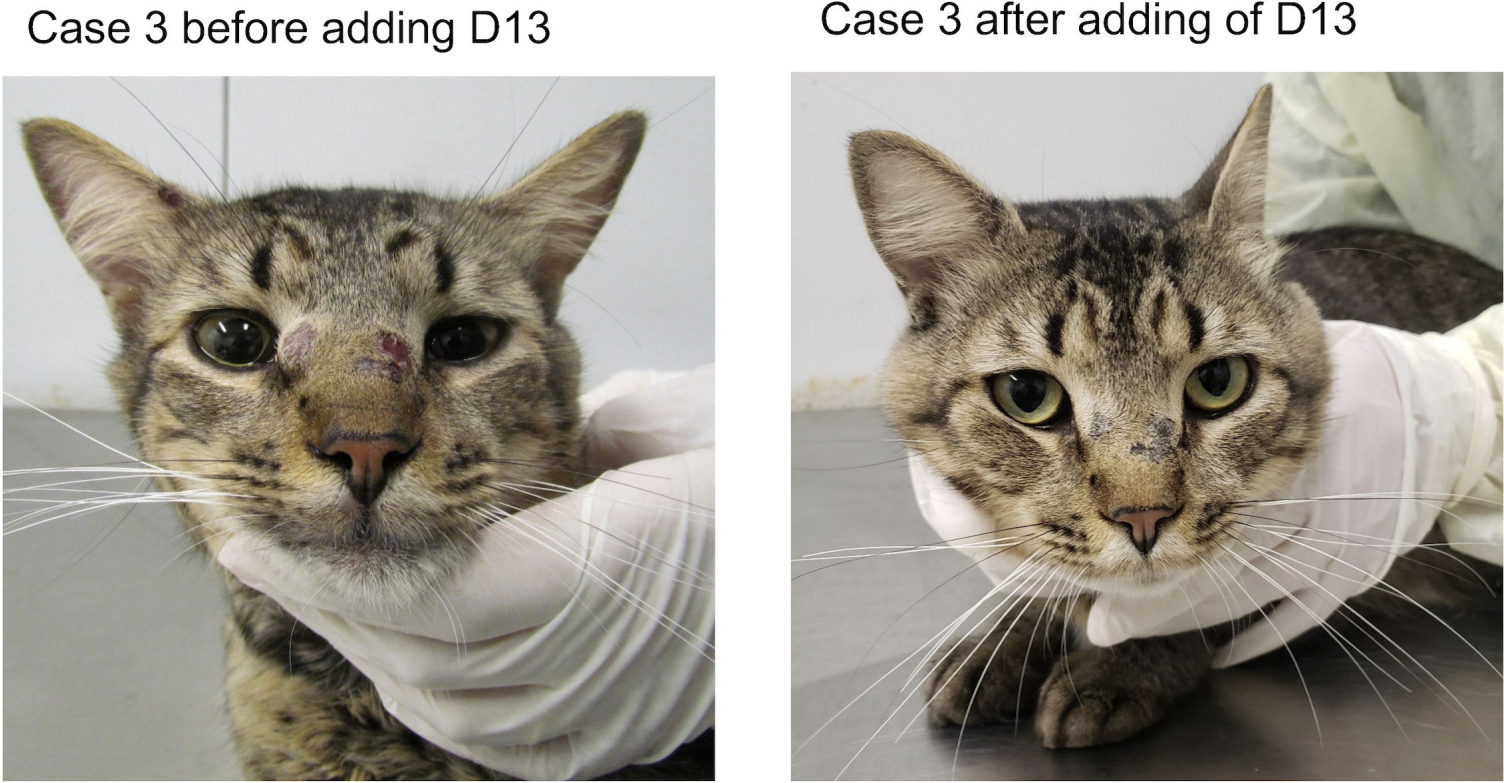
Case 3. The cat presented no improvement of the cutaneous and nasal mucosal lesions upon treatment with ITC. After adding D13, the cat significantly improved, and clinical cure was achieved after 12 weeks of treatment with ITC and D13 combination.

The patient was a 1-year-old non-neutered male crossbred cat weighing 4 kg. Clinical examination revealed multiple ulcerated, cutaneous lesions on the nasal bridge, lips, left ear, and right forelimb. Swelling of both nostrils, sneezing, nasal discharge, and enlarged mandibular lymph nodes were also observed. The cat was in good overall condition. ITC (100 mg/cat/day) was prescribed, and after 3 months of therapy, hyporexia was noted. Also, ITC failed to resolve the cutaneous lesion on the nasal bridge. In addition, during ITC treatment, three new lesions appeared: one on the nasal bridge near the other lesion and the other two on the right pinna. Thus, D13 (20 mg/kg/day) was added to ITC (100 mg/cat/day). During the 3 months of combination therapy, no clinical adverse reactions or abnormal laboratory findings were observed. After 30 days, mild-to-moderate elevations in ALT (ALT 103 U/L, reference interval 10–83 U/L) and at day 60 (ALT 266 U/L, reference interval 10–83 U/L) were noted. This abnormality in ALT level was transient and returned to the pre-treatment level when measured at day 90 of treatment. The clinical cure occurred after 3 months of combination therapy, when the cat was discharged (Fig. 5).

#### Case 4

**Fig 6:**
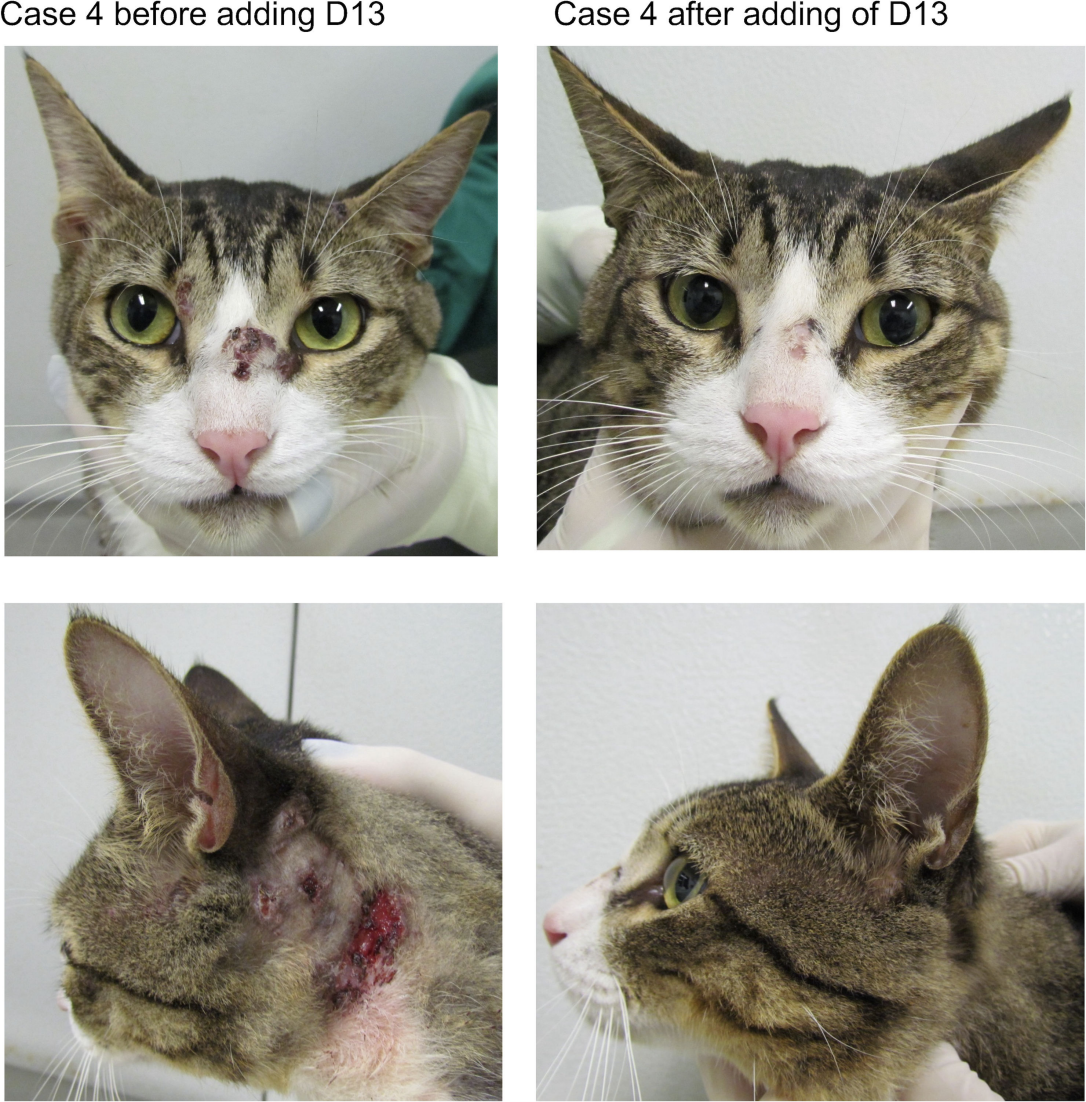
Case 4. The cat presented no improvement of the cutaneous lesions in the cephalic region and left cervical region upon treatment with ITC. After adding D13, the cat significantly improved, and clinical cure was achieved after 12 weeks of treatment with ITC and D13 combination.

The patient was a 2-year-old castrated male crossbred cat weighing 4.9 kg, and it was examined at the same reference center as above. The cat presented ulcerated, cutaneous lesions on the nasal bridge, left mandible, and dorsal thoracic region and one nodule in the medial corner of the left eye. An enlarged right mandibular lymph node was the only extracutaneous sign observed. The cat`s overall condition was good. ITC (100 mg/cat/day) was prescribed orally once a day. After 30 days of ITC treatment, the cat was evaluated. Although there were no clinical adverse reactions or abnormal laboratory findings, the cutaneous lesions persisted, indicating therapeutic failure. Thus, D13 at 20 mg/kg/day was added to ITC (100 mg/cat/day). The clinical cure occurred after 3 months of therapy, when the cat was discharged (Fig. 6). No clinical adverse reactions or abnormal laboratory findings were noted during the combination therapy.

#### Case 5

**Fig 7:**
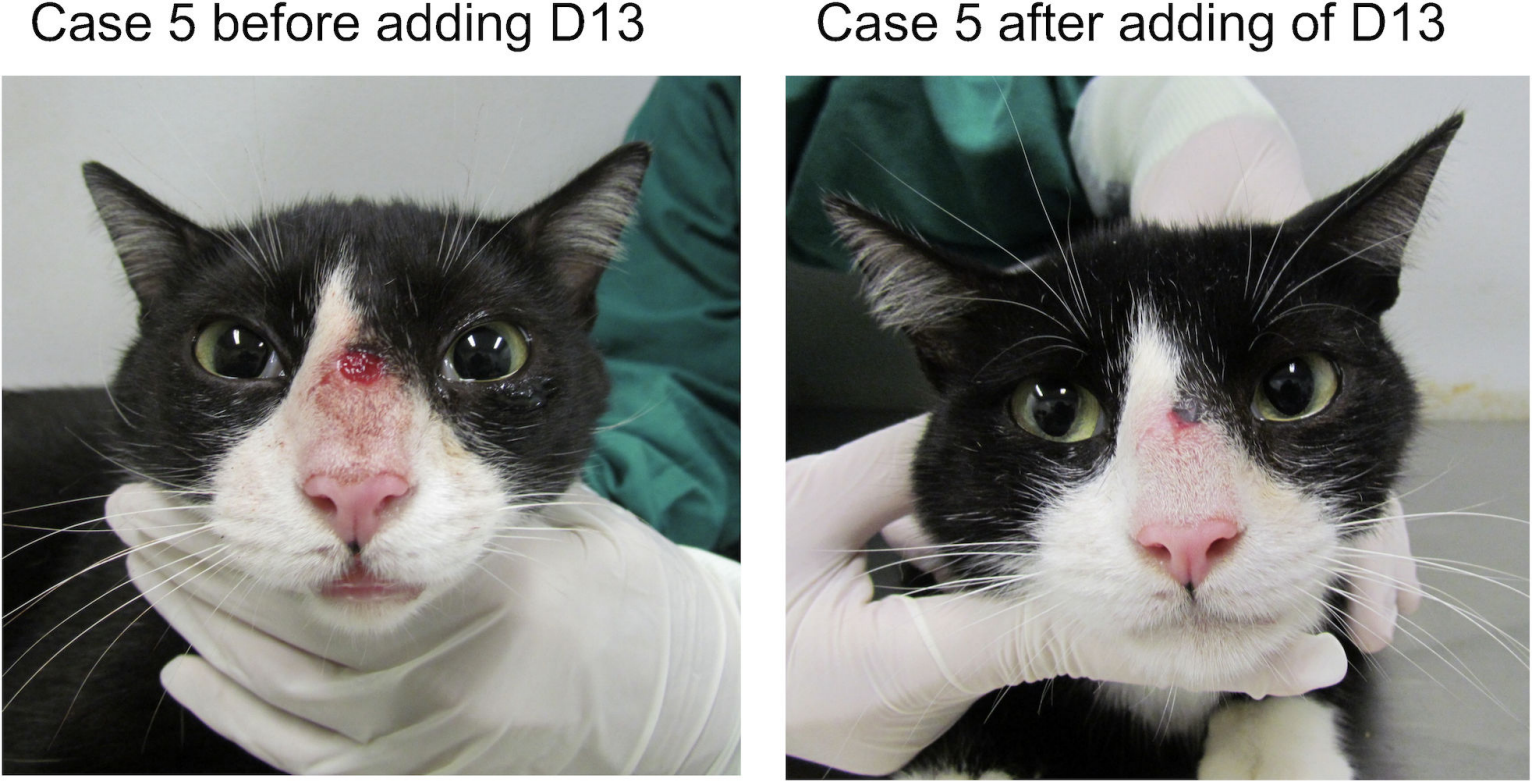
Case 5. The cat presented no improvement of the tumor-like lesion and of an ulcerated lesion on the nasal region upon treatment with ITC. After adding D13, the cat significantly improved, even though clinical cure was not achieved after 4 weeks of treatment with ITC and D13 combination.

The patient was a 3-year-old non-neutered male crossbred cat weighing 4.2 kg, and it was examined at the same reference center as above. The cat presented multiple ulcerated, cutaneous lesions on the nasal bridge, head, right forelimb, and tail. Swelling of both nostrils, sneezing, nasal discharge, and enlarged mandibular lymph nodes were also observed. The cat was in good overall condition. ITC (100 mg/cat/day) was prescribed, and after 4 weeks of therapy the persistence of the cutaneous lesions and the respiratory signs was observed. Thus, D13 (20 mg/kg/day) was added to ITC (100 mg/cat/day). During the 4 weeks of combination therapy, weight loss and mild elevation in ALT (ALT 112 U/L, reference interval 10–83 U/L) were observed. There was an improvement (Fig. 7) in the cutaneous lesions, but the respiratory signs persisted.

Among the other five included cats, all of them had cutaneous lesions and four had cutaneous and mucosal lesions. Respiratory signs (nasal discharge, sneezing, and dyspnea) were observed in four cats. Three cats had serious overall conditions. Unfortunately, three cats were lost during follow-up (treatment abandonment), whereas the other two cats had a progressive evolution of sporotrichosis. Both died after 8 weeks, and one of them died despite 72 weeks of treatment. No ALT elevation was observed in these five cats during the observation period.

## DISCUSSION

This is the first report evaluating the use of an acylhydrazone derivative (D13) in combination therapy with ITC for the treatment of feline sporotrichosis. Feline sporotrichosis is an important disease in Brazil, both for animal welfare reasons and for its zoonotic nature. ITC is the drug of choice, even if reports of therapeutic failure are common (11–14). The 10 cats reported here are typical cases of therapeutic failure for the SOC when cutaneous lesions are persisting despite the oral therapy with ITC.

When ITC fails as a monotherapy, it is normally associated with KI (11, 16). However, KI is not widely available, particularly in countries where feline sporotrichosis is endemic. Other options include the addition of AMB or TRB to ITC. However, intravenous (IV) AMB is toxic and TRB is expensive. Cryosurgery, intralipid AMB, or surgical removal of the lesions are other options with limited use, depending on the location of the lesion and the disadvantages of requiring sedation (16, 26). Thus, there is a need for alternative therapeutic options to overcome the inefficacy, toxicity, and drug resistance of the SOC.

Because the combination of ITC and D13 was shown to be effective *in vitro* and D13 was shown to be effective alone in a mouse model of sporotrichosis (23), we wondered whether D13 would also be effective in treating feline sporotrichosis. Contemplating this, we performed *in vitro* anti-fungal assays to test the *Sporothrix* clinical isolates from cats’ lesions exudate. Whereas not all clinical isolates were susceptible to ITC, all clinical isolates tested in our study were susceptible to D13. Furthermore, D13 was shown to be up to four times more potent than ITC against both planktonic cells and mature biofilm formed by *S. brasiliensis*. Importantly, synergistic studies showed that the clinical isolates were susceptible to the combination of ITC and D13, suggesting that the addition of D13 may re-sentisize the clinical isolates to ITC, as we observed previously with this class of new anti-fungals when combined with azoles (24, 27).

With these promising results, we submitted an animal protocol at Fiocruz with the goal to provide an alternative and effective strategy for the treatment of feline sporotrichosis. Our animal protocol was only approved as a compassionate drug use and only if D13 were to be added to the SOC, when the SOC did not improve any clinical signs of the cat. Precisely, failure of the SOC was defined as no improvement of skin lesion(s) or/and the appearance of new skin lesion(s) during at least 4 weeks of SOC alone.

Ten cats fitted these criteria and, upon consensus obtained from the owner, they were enrolled into the compassionate treatment regime that included D13. In one case, we saw improvement (Fig. 7), and in four cases (Fig. 3 to 6), we observed a complete and remarkable clinical cure of all lesions (Table 3). Two cats showed few mild clinical signs, such as hyporexia, nausea, and vomiting after the addition of D13. However, these signs were also observed during ITC treatment, particularly when ITC was combined with KI, as extensively reported (11, 28). Besides, gastrointestinal discomfort is common during sporotrichosis, which can result in a decrease of food intake. Thus, it is unlikely that these clinical signs are due to the addition of D13. Of note, because the drugs were administered alongside food, these clinical signs may explain the failure of the combination therapy in some cats. After all, drugs were administered with the food. However, the combination therapy with D13 was also not successful in three cats that did not suffer from nausea or vomiting. Lastly, since the food was given at home under the supervision of the owners, we are not sure if the cats consumed the complete dosage of the drugs each and every day.

Treatment success depends not only by a successful absorption of the drug administered but also by many additional factors, such as the overall health status, the susceptibility of the fungus to the drug, and the ability of the host immunity to counteract the fungal growth/survival in the host so that tissue sterilization can be achieved. We were indeed pleasantly surprised to see tissue sterilization of the cutaneous lesions in four cats.

The presence of nasal mucosa lesions and respiratory signs is a risk of therapeutic failure in cats. Three of our treated cats presenting respiratory signs did not respond to our combination therapy. In addition, cats with a serious overall condition may have disseminated sporotrichosis with internal organ lesions, in addition to cutaneous lesions, which are notoriously more difficult to treat (8, 13). Respiratory signs could be linked with deep organ infection and, in this case, it was too late for the addition of D13 to improve the overall condition. Notably, disseminated sporotrichosis is characterized by lesions with a high fungal load, and it is notoriously associated with a much longer healing time. If respiratory signs are present, therapeutic failure is common (7, 8). Moreover, the host immune response may also negatively influence the prognosis despite the therapeutic regime, although based on our lab analysis, the cats enrolled in this study did not show a low white blood cell count.

Re-exposure to fungal load is also an important issue. Upon the enrollment and during the treatment regimes (SOC alone or SOC + D13, these cats were not strictly kept in an isolation facility or isolated at home. If they were able to walk, they were free to go outside and interact with other cats. Most likely, some of these other cats were also affected by sporotrichosis, given the endemic nature of this disease among the cats in Rio do Janeiro. Consequently, our treated cats could have been re-exposed to additional fungal load from these other cats. In future studies, it will be important to keep the cats isolated, at least until the cutaneous lesions are completely healed.

Fungal resistance to ITC could also have been a factor, as the *S. brasiliensis* clinical strains isolated from the cats that succumbed to the infection were resistant to ITC (MIC_90_ ≥32 µg/mL). Even thought the combination of ITC with D13 was synergistic in some cases against these strains, perhaps this drug combination was administered too late and, in this case, did not improve the clinical signs of the disease.

As discussed above, gastrointestinal discomfort is also common during sporotrichosis. This may lead to a decrease of food intake. Because our drugs were introduced with food and the food was given at home, we are not sure if the cats assumed the full dose of the drugs everyday.

Future studies should also examine different formulations or/and different routes of administration of D13, such as IV) or intramuscular (IM), at least until the gastrointestinal discomfort diminishes or/and until the cat resumes its normal diet. This will ensure D13 will have its full anti-fungal effect. An IV or IM administration could also quickly improve the overall cat condition due to the fungicidal activity of D13. Improving the gastrointestinal discomfort will allow the administration of the drug(s) with food.

Whereas we did not observed any clinical adverse reactions when administering D13 to mice, we did notice an elevation of ALT in mice due to D13 (21). Therefore, in addition to ITC, the elevation of ALT in the four cats may be caused by D13. Please note, however, that similar to what was observed in mice (21), this elevation was mild and transitory with the normalization of the liver enzyme within 1 week from the discontinuation of D13.

Because of the nature of this pilot clinical study as compassionate drug use, it was not ethically possible to include a proper control, such as a group continuing the SOC (without D13) or a group taking placebo (no drugs). However, the remarkable success obtained in four cats when D13 was added to ITC should prompt the modification of the animal protocol and allow the administration of D13 as a monotherapy or as a combination therapy with ITC in naïve cats that have not received any anti-fungal prior to the initiation of D13. This animal protocol should include proper controls (e.g., animal treated with ITC ± KI or SOC alone). A partnership with industry to provide a large-scale synthesis of D13 is required to perform these studies.

The results obtained using the biofilm model of the cat claw is also intriguing. The ability of D13 to significantly reduce the biofilm formation in the claw may lead a reduction of the fungal load in the cat claw. As a result, transmission of the fungus from animal to animal or/and from animal to human should be reduced because the transmission mainly occur through scratches using the cat claw(s).

In conclusion, based on these preliminary results, the combination of ITC and D13 proved to be successful in a small number of cats with sporotrichosis refractory to ITC. In addition, D13 appeared to be safe and well tolerated with no or minor abnormal laboratory findings. A larger randomized trial is necessary to confirm the effectiveness and the safety of D13 as a monotherapy or/and in a combination treatment regime for feline sporotrichosis.

## MATERIALS AND METHODS

### Reagents

ITC (≥98% thin-layer chromatography) and RPMI 1640 [supplemented with L-glutamine, 2% (wt/vol)] of glucose and without sodium bicarbonate were purchased from Sigma-Aldrich, USA. Brain heart infusion (BHI) was acquired from Oxoid, Brazil.

### Large-scale synthesis of D13

Large-scale synthesis (1,000 g) of D13 (NED-59254–02) was commissioned and performed by New England Discovery Partner (NEDP), and a material safety data sheet was provided (see supplemental material). The compound was subjected to high-performance liquid chromatography (HPLC), liquid chromatography-mass spectrometry (LC-MS), and proton nuclear magnetic resonance (1H-NMR) analysis (data not shown). When shipped, the compound was placed in dry ice. Upon arrival, the powder was stored at −20C. Under this storage condition, D13 is active at least for 3 years. Small-scale synthesis of D13 (100 mg) was also performed in our laboratory. This small batch of D13 was then compared with the big batch of D13 (from NEDP) by HPLC, LC-MS, 1H-NMR, and by *in vitro* susceptibility analysis to make sure D13 from NEDP was identical in chemical structure and in activity to the D13 produced by our lab.

The drugs, as powder, were encapsulated. The capsules were given to the owner, who opened them on top of the cat wet food, and the drug powder was mixed in the food. This oral treatment was given daily and administered by the owner.

### *Sporothrix* strains and culture conditions

*Sporothrix brasiliensis* ATCC 5110/MYA4823 was obtained from the American Type Culture Collection (Manassas, VA, USA). All strains were cultured in BHI broth at 37°C with orbital agitation (150 rpm) for 3 days and then cryopreserved at −80°C in BHI broth with 20% glycerol.

Clinical isolates were obtained as follows. Exudates from a cutaneous or mucosal lesion were collected by impression smear on a glass slide for cytological examination. The slide was air-dried, stained using the Quick Panoptic method (Instant Prov Kit; Newprov, Pinhais, Brazil), and then analyzed under an optical microscope at ×1,000 magnification (29) of the presence of yeasts-like cells suggestive of *Sporothrix* spp. Exudates from the same lesion were also seeded on to Sabouraud dextrose agar and Mycobiotic agar (Difco) plates, incubated at 25°C for up to 4 weeks for fungal growth. Suspected isolates were sub-cultured on potato dextrose agar medium (Difco) at 25°C for macroscopic and microscopic morphological studies. Dimorphism was demonstrated by conversion to the yeast-like form on brain heart infusion agar medium (Difco) at 37°C. *Sporothrix* spp. was isolated in mycological culture, and *S. brasiliensis* was identified using the molecular tool T3B PCR fingerprinting (30). *Sporothrix brasiliensis* was identified in 8 out of 10 cats. From two cats, we were not able to identify the fungal species because the samples were highly contaminated by bacteria.

### Anti-*Sporothrix* tests

Anti-fungal assays were conducted according to the Clinical and Laboratory Standards Institute M27-A3 protocol, with necessary adjustments for cell suspension. Yeasts were cultured in BHI broth at 37°C/150 rpm for 3 days. Subsequently, the cells were washed in phosphate-buffered saline (PBS) and adjusted to a concentration of 5 × 10^5^ yeasts/mL in RPMI 1640. D13 and ITC were diluted in RPMI ranging from 16.0 to 0.06 µg/mL, and 100-µL volumes were added to 96-well plates. Then, 100 µL of the yeast suspension was added to each well. Cells were incubated at 37°C for 3 days. RPMI 1640 was used as control. Fungal growth was quantified using a BioTeK ELx808 microplate reader (Netherlands) through spectrophotometric measurements taken at a wavelength of 530 nm. The minimal inhibitory concentrations (MIC_50_ and MIC_90_) were determined as the lowest concentrations of D13 that reduced microbial growth by 50% and 90%, respectively, compared to the control conditions.

### Combination drugs

The experimental procedure was conducted using 96-well plates, following the checkerboard test outlined in previous studies (23). Succinctly, serial twofold dilutions of each drug (D13 and ITC) were prepared in RPMI. Yeasts (5 × 10^5^ cells/mL) was added into the 96-well microplates in 100-µL volumes, after which the plates were incubated at 37°C for 3 days. The optical density was measured at 530 nm using a BioTek ELx808 microplate reader. The fractional inhibitory concentration index (FICi) was calculated as (MIC combined/MIC drug A alone) + (MIC combined/MIC drug B alone). Synergistic drug interactions were identified as synergistic when the FICi was less than 0.5, indifferent interactions when the FICi was between 0.5 and 4.0, and antagonistic interactions when the FICi exceeded 4 (23, 31, 32). Alternatively, we calculated the Bliss synergistic scores using the SynergyFinder tool (33). A score below −10 was interpreted as an antagonistic interaction, while scores ranging from −10 to 10 indicated an additive interaction. Scores greater than 10 denoted a synergistic interaction between the two drugs.

### *Ex vivo* biofilm

The anti-biofilm effect of D13 was visualized by SEM and quantified by CFU determination. Claws collected from healthy cats were previously sterilized by immersion in 90% ethanol/3 h, washed 3× with PBS, and transferred to tubes with 200 µL of RPMI/1% PenStrep containing 1 × 10^6^ cells/mL of *S. brasiliensis* ATCC 5110. Cells were incubated at 37°C for 3 days. Afterward, claws were washed 3× with PBS in order to remove the cells not adhered to the biofilm. The biofilm formation was checked using an optical microscope (Olympus CX-31), and then 200 µL of D13 (10 µg/mL) or ITC (10 µg/mL) previously diluted in RPMI 1640 was added. After 3 days of incubation at 37°C, the claws were washed with PBS, and the cells were fixed with 2.5% glutaraldehyde and 4% formaldehyde (in 0.1-M cacodylate buffer, pH 7.2) for 1 hour at room temperature. The claws were then washed with cacodylate buffer and dehydrated using ethanol gradient. Samples were coated with a 20-nm layer of gold and visualized using a scanning electron microscope (ZEISS EVO MA 10, Germany) operated at 10 kV.

To determine the CFU, claws from each treatment and control were collected and weighed prior to the fixation stage. The claws were suspended in 200 µL of RPMI 1640/1% PenStrep, subjected to sonication for 1 minute, and vortexed. Aliquots of 20 µL were plated onto BHI plates. Following an incubation of 2–3 days at 37°C, the CFU count was performed. Two independent experiments were conducted.

### Statistical analysis

Data were analyzed using GraphPad Prism version 7 software, employing ordinary one-way analysis of variance followed by Tukey’s multiple comparisons test. Statistical significance was determined by a *P* value of less than 0.05.

## SUPPLEMENTAL MATERIAL

Supplemental material spectrum.03967-23-s0001.pdf MSDS of D13.

ASM does not own the copyrights to Supplemental Material that may be linked to, or accessed through, an article. The authors have granted ASM a non-exclusive, world-wide license to publish the Supplemental Material files. Please contact the corresponding author directly for reuse.
