# Supplementary material for: Combination therapy of itraconazole and an acylhydrazone derivative (D13) for the treatment of sporotrichosis in cats
Source: Microbiol Spectr. 2024 Apr 22;12(6):e03967-23. doi: 10.1128/spectrum.03967-23 (PMC11237696; doi:10.1128/spectrum.03967-23)
Supplement: Supplemental material — MSDS of D13. [file spectrum.03967-23-s0001.pdf]

New England Discovery Partners

MATERIAL SAFETY DATA SHEET

SECTION 1.——————— CHEMICAL IDENTIFICATION ————————

Name: NED 59254; (E)-4-bromo-N'-(3,5-dibromo-2-hydroxybenzylidene)benzohydrazide

SECTION 2.———COMPOSITION/INFORMATION ON INGREDIENTS————

Molecular Formula: $C_{14}H_9Br_3N_2O_2$
Mol. Wt.: 476.95

SECTION 3.——————————HAZARDS IDENTIFICATION—————————

LABEL PRECAUTIONARY STATEMENTS

In case of contact with eyes, rinse immediately with plenty of water and seek medical advice.

Wear suitable gloves and eye/face protection and dust mask.

SECTION 4.——————————FIRST AID MEASURES———————————

In case of contact, immediately flush eyes with copious amounts of water for at least 15 minutes.

In case of contact, immediately wash skin with soap and copious amounts of water.

If inhaled, remove to fresh air. If not breathing give artificial respiration. If breathing is difficult, give oxygen.

If swallowed, wash out mouth with water provided person is conscious. Call a physician.

Wash contaminated clothing before reuse.

SECTION 5.————————FIRE FIGHTING MEASURES——————————

EXTINGUISHING MEDIA

Water spray

Carbon dioxide, dry chemical powder or appropriate foam

SPECIAL FIRE FIGHTING PROCEDURES

Wear self-contained breathing apparatus and protective clothing to prevent contact with skin and eyes.

NEDP
New England Discovery Partners

SECTION 6.————————ACCIDENTAL RELEASE MEASURES——————————

Wear heavy rubber gloves.

Wear protective clothing and dust mask.

Sweep up, place in a bag and hold for waste disposal.

Avoid raising dust.

Ventilate area and wash spill site after material pickup is complete.

SECTION 7.—————————HANDLING AND STORAGE————————————

Store in glass bottles or bags protected from light at room temperature.

SECTION 8.————EXPOSURE CONTROLS/PERSONAL PROTECTION————

Chemical safety goggles

Rubber gloves

Dust mask

Safety shower and eye bath

Mechanical exhaust required.

Avoid contact and inhalation.

Do not get in eyes, on skin, or clothing.

Wash thoroughly after handling.

Irritant

Keep tightly closed.

Store in a cool dry place

SECTION 9.———————PHYSICAL CHEMICAL PROPERTIES————————

APPEARANCE AND ODOR: white powder

SECTION 10.—————————STABILITY AND REACTIVITY——————————

INCOMPATIBILITIES

Strong oxidizing agents, Strong acid or base

HAZARDOUS COMBUSTION OR DECOMPOSITION PRODUCTS

Toxic fumes of:

Carbon monoxide, carbon dioxide, nitrogen oxide

SECTION 11.————————TOXICOLOGICAL INFORMATION————————

ACUTE EFFECTS

The complete toxicological effects of this compound in animals or humans have not yet been adequately investigated.

SECTION 12.——————————ECOLOGICAL INFORMATION—————————

Data not yet available

SECTION 13.——————————DISPOSAL CONSIDERATIONS——————————

Dissolve or mix the material with a combustible solvent and burn in a chemical incinerator equipped with an afterburner and scrubber.

Observe all federal, state and local environmental regulations.

SECTION 14.——————————TRANSPORT INFORMATION———————————

Data not available

SECTION 15.——————————REGULATORY INFORMATION—————————

Data not available

SECTION 16.————————————OTHER INFORMATION———————————

The above information is believed to be correct but does not purport to be all inclusive and shall be used only as a guide. The Institutes for Pharmaceutical Discovery, LLC (IPD) shall not be held liable for any damages resulting from handling or from contact with the above product. In case of an emergency, contact Michael Van Zandt at Phone # 203-494-1672.
